# Systemic Levamisole-Induced Vasculitis in a Cocaine User without Cutaneous Findings: A Consideration in Diagnosis

**DOI:** 10.1155/2015/547023

**Published:** 2015-10-08

**Authors:** Gillian G. Baptiste, Anastasia-Stefania Alexopoulos, Tahsin Masud, Joanna M. Bonsall

**Affiliations:** ^1^Department of Medicine, Emory University, Atlanta, GA 30308, USA; ^2^Division of Renal Medicine, Department of Medicine, Emory University, Atlanta, GA 30308, USA; ^3^Division of Hospital Medicine, Department of Medicine, Emory University, Atlanta, GA 30308, USA

## Abstract

Levamisole is a known immunomodulating agent frequently used as a cutting agent in cocaine consumed in the United States today. Numerous cases of anti-neutrophil cytoplasmic antibody (ANCA) vasculitis connected with the use of levamisole-adulterated cocaine have previously been reported in the literature, classically characterized by a retiform purpuric rash. We report a case of a crack-cocaine user without cutaneous abnormalities who developed ANCA-associated glomerulonephritis that progressed to renal failure. This case demonstrates the difficulties in solidifying the diagnosis of levamisole-induced vasculitis in the absence of cutaneous findings and the need to pursue more testing to establish causality in ANCA-associated vasculitis that has potential for severe end-organ damage in patients who continue to use cocaine.

## 1. Introduction

It is estimated that over 70% of cocaine consumed in the United States today is contaminated with levamisole, a veterinary antihelminthic agent known for its immunomodulator effects. Levamisole is used as a cutting agent in cocaine both to decrease costs and to improve the euphoric effects of cocaine on the brain [1, 2]. With the rise of the rate of levamisole adulteration of cocaine over the past decade, an increasing number of cases of anti-neutrophil cytoplasmic antibody (ANCA) vasculitis have been connected back to cocaine use [3].

Levamisole-induced vasculitis has classically been characterized by a retiform purpuric rash, in addition to ANCA positivity [4, 5]. Herein we describe a case of a crack-cocaine user without cutaneous abnormalities who developed ANCA-associated glomerulonephritis that progressed to renal failure. This case demonstrates the potential for severe end-organ damage in patients with levamisole-induced ANCA vasculitis and the need for high clinical suspicion in cocaine users with any findings suggestive of vasculitis.

## 2. Case Presentation

A 43-year-old homeless male presented with several weeks of weight loss, bilateral burning foot pain, and overall weakness that had progressed to an inability to walk. The past medical history was largely unknown but included admission to a different hospital two years prior with acute kidney injury secondary to rhabdomyolysis, with a serum creatinine of 1.5 mg/dL on discharge. The social history was significant for crack-cocaine use. On admission, he appeared cachectic and had a temperature of 38.7°C. On the neurological exam, he had impaired soft touch sensation bilaterally up to the ankles and diffuse weakness. There were no skin abnormalities seen.

**Figure 1:**
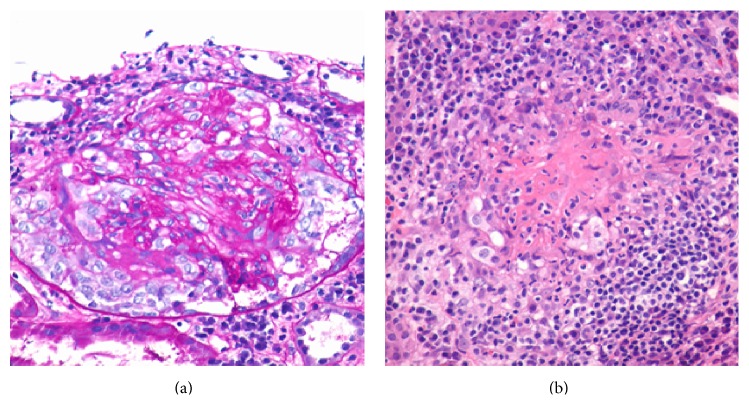
Renal biopsy. (a) PAS at 400x. Glomerulus showing proliferative glomerulonephritis surrounded by a cellular crescent. (b) H&E at 400x. Focus of fibrinoid necrosis with inflammation from necrotizing arteritis. PAS: periodic acid-Schiff-diastase; H&E: hematoxylin and eosin.

The creatinine on admission was 3.93 mg/dL, and the BUN was 55 mg/dL (upper limit of normal is 1.0 mg/dL and 25 mg/dL, resp.). A globulin gap was present with a total protein of 6.7 g/dL and albumin of 1.4 g/dL. Urinalysis demonstrated moderate protein and blood, with a spot protein-creatinine ratio of 1 : 55 mg/mg (upper limit of normal is 0.11 mg/mg). There was no peripheral eosinophilia. Microscopic urinalysis showed granular casts but no red blood cell casts. His urine tested positive for eosinophils. Cocaine metabolites were found on urine toxicology. HIV, anti-nuclear and anti-glomerular basement membrane antibodies, RPR, and hepatitis B and C serologies were negative. There were no abnormal bands on serum or urine protein electrophoresis. ANCA were strongly positive at 1 : 5120 (upper limit of normal is less than 1 : 20) with a perinuclear staining pattern and myeloperoxidase reactivity at 76 AU/mls (upper limit of normal is less than 19 AU/mls). Kidney biopsy specimen had a total of 21 glomeruli, out of which only two were globally sclerotic. The majority of glomeruli showed pauci-immune necrotizing crescentic glomerulonephritis (Figure 1). A prominent plasma cell-rich tubulointerstitial nephritis was also present.

Based on his laboratory findings and renal biopsy, a diagnosis of microscopic polyangiitis was considered, but levamisole-induced ANCA vasculitis remained a likely possibility given his history of cocaine use. The presence of interstitial nephritis on biopsy was felt to explain the eosinophils in his urine, a finding previously reported in cocaine users [6]. He was treated with pulse methylprednisolone 1000 mg for three days and 600 mg of IV cyclophosphamide. His creatinine peaked to 7.21 mg/dL during hospitalization but stabilized at 6.25 mg/dL after treatment. The patient was counseled on abstaining from cocaine and was discharged on 40 mg of prednisone per day with instructions to follow-up for monthly cyclophosphamide injections at renal clinic.

The patient was readmitted one month later with increasing lower extremity swelling and pain. He had been off prednisone since discharge and had ongoing crack-cocaine use. Renal function had declined, reaching serum creatinine of 8.8 mg/dL. He was treated with cyclophosphamide and prednisone, and his fluid status and electrolyte abnormalities were corrected. Renal function did not improve with treatment, and hemodialysis was initiated.

## 3. Discussion

The high prevalence of levamisole as an adulterant in cocaine and the number of cocaine users in the United States today make the diagnosis and treatment of levamisole-induced ANCA vasculitis an increasingly significant public health concern. Previous reports of levamisole-induced ANCA-positive disease have primarily noted cutaneous findings. Graf et al. reported on six cases of retiform purpura that progressed to necrosis and ulceration of the skin following exposure to levamisole-adulterated cocaine [7]. Such cutaneous findings, particularly purpuric lesions of the ears, are commonly considered classic manifestations of levamisole-induced vasculitis.

As awareness of levamisole-induced ANCA vasculitis from cocaine use has grown, cases with increasingly varied clinical presentations have been recognized. McGrath et al. reviewed 30 such cases of ANCA-positive disease in cocaine users. The most common clinical manifestations were arthralgias, constitutional symptoms, and skin lesions. Of note, 39% of patients had no abnormal cutaneous findings, indicating that typical purpuric lesions cannot be relied upon to make the diagnosis of levamisole-induced vasculitis [8]. All cases had anti-myeloperoxidase antibodies, and 50% also showed anti-proteinase 3 antibodies [8]. Acute kidney injury was seen in two patients with combined positivity for anti-myeloperoxidase and anti-proteinase 3 antibodies, though neither patient progressed to the point of requiring renal replacement therapy.

Distinguishing levamisole-induced ANCA vasculitis from autoimmune ANCA vasculitis remains a challenge. Detecting levamisole in serum or urine requires gas or liquid chromatography-tandem mass spectrometry, which is often not easily accessible. Furthermore, the plasma half-life of levamisole is estimated at only 5.6 hours [9]. Following their series in which only 12.5% of patients tested positive for levamisole, Gross et al. proposed that a positive test for levamisole should not be required to make the diagnosis of levamisole-induced vasculitis in patients with the characteristic clinical presentation [10]. Other biomarkers have been sought as alternatives to support the diagnosis of levamisole-induced vasculitis. For example, ANCA positivity with specificity for human neutrophil elastase was reported to distinguish levamisole-induced vasculitis from autoimmune vasculitis [3]. However, it remains unclear whether human neutrophil elastase antibodies are a marker of levamisole-induced vasculitis or simply of cocaine use, and this test is also not readily available at most centers.

In the absence of cutaneous findings, this case highlights the challenges encountered in solidifying the diagnosis of levamisole-induced vasculitis due to variable presentation and difficulty obtaining confirmatory laboratory tests. Without firm evidence on the efficacy of systemic corticosteroids for levamisole-induced vasculitis patients, it is imperative that a combination of supportive care and counseling on cocaine cessation be the cornerstone of treatment. Suspicion for levamisole-induced ANCA vasculitis in cocaine users with any vasculitic findings should be high, and such patients should be treated and counseled appropriately in an effort to prevent further progression of disease.
